# Aniline Poisoning and Its Treatment

**Published:** 1910-04-02

**Authors:** 


### Toxicology.

### ANILINE POISONING AND ITS TREATMENT.

Both the symptoms, and the importance of persistence with the oxygen treatment, of cases of aniline poisoning are exemplified by the following case. The patient was a strong man, aged 48, who on July 14, at about 11.30 a.m., had drunk, in mistake for ale, about three ounces of aniline sulphite solution, made by dissolving three handfuls of the crystals in a pail of water. This solution is used for testing purposes in the manufacture of paper. Noticing the peculiar taste, he rinsed his mouth immediately, and then proceeded with his work. Twenty minutes later his fellow-workmen remarked that he was looking ill, but it was not until another 30 minutes had elapsed that he himself noticed any symptoms. He then became giddy and weak, his heart palpitated, and he sweated profusely. He then fell down, but did not lose consciousness. He had no pain whatever. His companions gave him salt solution, which caused vomiting on his way to hospital.

On admission the patient had a very livid grey colour, and he was rapidly becoming weaker. His stomach was at once washed out, oxygen was administered continuously from a mask, and a pint of normal saline solution was infused into each axilla. The patient was quite comfortable, and there was no dyspnœa; his temperature was 99° F., his respiration rate 24, and his pulse 114. The blood, which was chocolate brown in colour, contained methæmoglobin. Histologically, there was nothing abnormal in the appearance of the red cells. At 2 p.m. the patient's colour showed slight improvement; his pulse was good, but his respiration was rather shallow. At 3 p.m. oxygen was given per rectum in addition to that administered from the mask. Half an hour later the man became rapidly more drowsy, especially during the few seconds that oxygen was not given owing to changing the cylinder. Cheyne-Stokes respiration developed, but the pulse was still good.

### Progress of the Case.

At 4 p.m. the patient was deeply comatose, and his respirations were very shallow, but there was no dyspnœa. A few minutes later spontaneous respiration ceased. Artificial respiration was performed, and normal respiratory movements soon returned, but the patient was still comatose.

At 5 p.m. breathing was better. The patient's grey colour had not changed in the slightest degree since 2 p.m. He was now given a minim of croton oil in milk by means of a nasal tube.

At 6 p.m. he was still comatose, the pulse rate being 84, the respiration rate 28, and the temperature 103°.

At 9 p.m. he had a nasal feed of milk, egg, and brandy.

At 10 p.m. he was still comatose.

At 11 p.m. he was rather restless, and frequently moved his head in order to throw off the oxygen mask. On being told to remain quiet, he showed signs of consciousness for the first time since 3.30 p.m.

His breathing had much improved by midnight, and at 1 a.m. next morning his colour began to improve. Respiration was excellent.

At 2 a.m. he was more restless. The pulse and respiration rates were normal and the temperature 100° F. Oxygen was no longer administered. The colour was much the same as at 1 p.m. During the rest of the night the patient's condition gradually improved, and at 9 a.m. his colour was normal. He now slept naturally, and afterwards was quite conscious. His blood now contained 80 per cent. hæmoglobin, and no methæmoglobin was present. The croton oil had caused five evacuations of the bowels, and the stools were quite chocolate in colour, suggesting the presence of a large amount of methæmoglobin. The urine was normal, no methæmoglobin being excreted in it.

On the 17th he was allowed to get up, and on the 18th he left the hospital quite well. A week later he reported that he was quite well, in his usual state of health.

### Some Conclusions.

The following important conclusions can be drawn from this case:—

1. The symptoms of aniline poisoning are due to defective oxygenation of the tissues from the conversion of oxy-hæmoglobin into methæmoglobin.
2. Methæmoglobin is reconverted into oxy-hæmoglobin within the body much more rapidly than physiological experiment would lead one to expect.
3. The red corpuscles are unaffected, no methæmoglobin being discharged into the blood-plasma or excreted in the urine.
4. The experiments of Haldane, showing that an animal that has not sufficient hæmoglobin left to support life in air can still live in an atmosphere of pure oxygen, are shown to apply with equal force to similar conditions in man. In the arterial blood of a man breathing air there is about 18.5 per cent. oxygen in combination with hæmoglobin, and 0.6 per cent. in simple solution. When pure oxygen is breathed there is about 18.7 per cent. in combination and 3 per cent. in solution. The oxygen in simple solution is much more easily used by the tissues than that in combination, so that in an atmosphere of pure oxygen the amount of easily available oxygen is largely increased, and is sufficient to maintain life even when the quantity in combination is very greatly reduced.
5. From conclusions 2 and 4 it follows that in all cases of poisoning by aniline, nitrites, etc., in which methæmoglobinæmia occurs, the continuous administration of pure oxygen by mouth and rectum, and, if necessary, artificial respiration, should be employed for several hours without intermission, as recovery will almost certainly occur, even if the patient, as in this case, appears moribund.

